# Entrepreneurial Self-Efficacy Mediates the Impact of the Post-pandemic Entrepreneurship Environment on College Students’ Entrepreneurial Intention

**DOI:** 10.3389/fpsyg.2021.643184

**Published:** 2021-04-28

**Authors:** Jiping Zhang, Jianhao Huang

**Affiliations:** ^1^The Office of Psychological Counselor, College of Foreign Languages, Ningbo University, Ningbo, China; ^2^Department of Education Management, China-Asean International College, Dhurakij Pundit University, Bangkok, Thailand

**Keywords:** entrepreneurial self-efficacy, COVID-19, entrepreneurial intention, entrepreneurial environment, post-pandemic

## Abstract

The mechanism of how the COVID-19 global pandemic has affected the entrepreneurial intentions of college students remains unknown. To investigate the impact of the entrepreneurial environment on entrepreneurial self-efficacy and entrepreneurial intentions in the post-pandemic era, 913 college students were invited to complete a questionnaire. The data were analyzed with structural equation models. The conclusions revealed by the questionnaire are as followed: college students have retained some entrepreneurial intention in the post-pandemic era; the factors influencing the entrepreneurial intention include sex, family entrepreneurial history, major, and education background; and entrepreneurial self-efficacy can play a major role to mediate the impact caused by the post-pandemic entrepreneurial environment on entrepreneurial intentions. The research conclusions provide important insights to improve college students’ entrepreneurial intentions in the post-pandemic environment.

## Introduction

The world witnessed the unprecedented epidemic of COVID-19 in 2020, which brought a huge shock to the global economy, trade, as well as investment. Major economies around the world successively experienced significant declines in the economic growth of varying degrees, accompanied by a sharp spike in unemployment and deterioration in the performance of the market (China Macroeconomic Forum, 2020). Therefore, economic recovery after the epidemic is becoming the top priority for any country. According to the *Global Competitiveness Report*, entrepreneurship, which is a momentum for a country to promote economic development, plays an irreplaceable role in maintaining social stability and achieving economic recovery in efficiency-driven countries. The reform of innovation and entrepreneurship education in higher education plays a critical role in the country’s socio-economic development, which in turn influences the international status of a country. Therefore, countries around the world attach great importance to the cultivation of entrepreneurial intentions (EI) among college students. In 2015, China’s State Council issued “Opinions on Several Policy Measures to Vigorously Promote Mass Entrepreneurship and Innovation,” putting forward “Mass Entrepreneurship and Innovation.” As college students are the reserve force for national development, study on their entrepreneurial intentions in the post-epidemic environment (PEE) is vital to the country’s economic recovery.

It has been found that the entrepreneurial environment is a significant positive predictor of entrepreneurial intentions (Wu and Mao, 2020). Essel et al. (2020) have found that a good entrepreneurial environment promotes entrepreneurial intentions with empirical studies. Also, some scholars have found a decline in social entrepreneurial intentions due to the epidemic. For example, Inés Ruiz-Rosa et al. (2020) have found that the epidemic caused a severe socio-economic crisis and great uncertainty, which led to a decline in students’ entrepreneurial intentions. However, the post-epidemic entrepreneurial environment has been changed dramatically. Under the epidemic prevention and control guidelines, people in China and beyond has experienced online activities like online shopping and online education, and people’s mindset and lifestyles changed at the same time, which is more embracing a new lifestyle with the internet economy (Wang et al., 2020; Tang and Liang, 2021). Although the epidemic has brought a severe blow to the service industries such as transportation, travel, and retail. But online consumption such as telecommuting, telemedicine, online education, online entertainment, and emerging industries such as unmanned delivery and smart manufacturing have all shown strong potential to grow (Duan, 2020). Be that as it may, there is no sound evidence on how exactly the post-epidemic entrepreneurial environment predicts entrepreneurial intentions.

However, currently, many countries are under the pressure of the epidemic, and when it is under control and becomes stable, it is an urgent need to create a good entrepreneurial environment to boost people’s entrepreneurial intentions. China has taken the lead in controlling the epidemic, and as of 27 September 2020, all 31 provinces (autonomous regions and municipalities) and the Xinjiang Production and Construction Corps have resumed cross-provincial (autonomous regions and municipalities) group travels and entered the post-epidemic era (Babna, 2020). General Secretary Xi Jinping pointed out: “The epidemic is both a challenge and an opportunity for industrial development. We need to take it as an opportunity to transform and upgrade traditional industries and innovate new industries.” In the post-epidemic situation, the Chinese government has already announced many entrepreneurship-friendly policies. Through the review of literature, it has been found that there is no precedent research on how entrepreneurial intentions are changed in the post-epidemic environment and how the entrepreneurial environment affects entrepreneurial intentions, and a possible reason behind this may be that most countries are still in the midst of the epidemic. But now China is embracing a post-epidemic era, and relevant studies are of great value and innovative significance.

Meanwhile, entrepreneurial self-efficacy (ESE), which refers to self-efficacy in the context of entrepreneurship, has been a key topic in research on entrepreneurship, which draws the attention of many scholars. In fact, entrepreneurial self-efficacy is dual; in that, it is both a mediating variable and an outcome variable. In this research, entrepreneurial self-efficacy is considered as a key mediating variable in the relationship between entrepreneurial environment and entrepreneurial intention in the post-pandemic era. With this in mind, this study also focuses on the relationship between entrepreneurial environment and entrepreneurial intention and investigates whether entrepreneurial self-efficacy can play a mediating role.

In summary, based on the literature review, this study finds that there is no precedent research conducted, even though there is sound evidence that entrepreneurial environment can predict entrepreneurial intentions, and entrepreneurial self-efficacy can bring a mediating effect in entrepreneurship. Given that China is among the first to enter the post-epidemic era, which can be considered as a sound representative. As a result, this study chooses China to study how the post-epidemic entrepreneurial environment (PEE) affects entrepreneurial intentions, which is both innovative and practical.

## Literature Review and Hypothesis Development

### The Theory of Social Cognitive Model

Bandura’s (1977) Social Cognitive Theory suggests that entrepreneurial intentions are deliberate, rational behavioral tendencies, which are the combined result of the entrepreneurial environment, entrepreneurial cognition, and individual-environment interactions. The theory emphasizes the influence of the social environment, in which the individual lives, on people’s behavior, pointing that the external environment can act as a resource for the individual to enhance self-prediction, manipulation, or volitional control. Thus, it can provide the individual with precise information that affects the direction and intensity of his or her behavior. At the same time, the influencing process of the external environment on behavior varies according to the individual’s cognitive knowledge and characteristics. In other words, the external environment can influence human behavior with various stimuli, and this influence is realized through the psychological process and internal self-regulatory system (i.e., self-efficacy) of an individual. Therefore, according to Social Cognitive Theory, the entrepreneurial environment can be viewed as the external environment, and entrepreneurial self-efficacy can be viewed as the individual’s cognitive knowledge and characteristics. Both of them can play major roles in the forming of an individual’s entrepreneurial intentions and are vital variables in the study on entrepreneurial intentions. The entrepreneurial environment can affect entrepreneurial intentions through individual judgments on the accessibility to resources and support.

### Entrepreneurship Intention

Entrepreneurial intention is defined as “a self-acknowledged conviction by a person that they intend to set up a new business venture and consciously plan to do so in the future” (Thompson, 2009). Similar to personal commitment, the Entrepreneurial intention of college students can predict the possibility of whether they will start a business in the future or not (Liu, 2011). As a consequence, this study regards entrepreneurial intentions as the behavioral dispositions and ideas of college students who choose to start their own business in the future.

### Linking Post-pandemic Entrepreneurial Environment to Entrepreneurial Intention

The entrepreneurial environment, which includes all social, cultural, economic, and political conditions, as well as existing opportunities to receive entrepreneurial resources, is a vital combination of factors that influences entrepreneurial activities (Zhang and Chen, 2004). The entrepreneurial intention model proposed by Lüthje and Franke (2010) indicates that the influence of social-environmental factors is vital for an individual’s entrepreneurial intention, that is to say, whether, at school, or society, environmental factors that can hinder entrepreneurship will frustrate an individual’s entrepreneurial intentions. Previous research has found that the entrepreneurial environment has a substantial impact on entrepreneurial intentions (Rajib and Niladri, 2020; Shi et al., 2020). To date, the entrepreneurial environment in China has changed with the outbreak of the global pandemic, but there has been little research on whether the post-pandemic entrepreneurial environment can affect the entrepreneurial intentions of college students. Thus, this study proposes a hypothesis based on previous research:

Hypothesis 1: The post-pandemic entrepreneurial environment has a substantial effect on student’s entrepreneurial intention.

### The Mediating Role Played by Entrepreneurial Self-Efficacy

Bandura (1977) has first introduced the concept of self-efficacy from a psychological perspective and defined it as people’s beliefs in their capabilities to do a job. Boyd and Vozikis (1994) have defined entrepreneurial self-efficacy as the belief that people are confident to become an entrepreneur, engage in entrepreneurial activities, and ultimately achieve their entrepreneurial goals. Self-efficacy determines one’s orientation of goals, the effort paid to achieve goals, and persistence. As a psychological resource, Self-efficacy has a positive impact on one’s behavior. Research has disclosed that entrepreneurial self-efficacy can play a mediating role in the relationship between multiple network embedding and entrepreneurial intention (Li et al., 2019). Also, it can moderate the relationship between extraversion, openness, emotional stability, and entrepreneurial intention (Jin and Huang, 2019). According to the Social Cognitive Theory, both the entrepreneurial environment and entrepreneurial self-efficacy are momentous in the formation of entrepreneurial intentions. Farashah (2015) has studied the institutional normative environment of 54 countries (regions), observing that the entrepreneurial self-efficacy of adults had a positive effect on individual entrepreneurial intentions; while a recent empirical study from Saudi Arabia has shown a partially mediating role played by entrepreneurial self-efficacy in the relationship between entrepreneurial environment and entrepreneurial intentions (Elnadi and Gheith, 2021).

Based on a thorough literature review, the relationship between the entrepreneurial environment, entrepreneurial self-efficacy, and college students’ entrepreneurial intention was assessed in the post-pandemic era. Specifically, the direct effect of the post-pandemic entrepreneurial environment on entrepreneurial intention was tested and whether entrepreneurial self-efficacy can play a mediating role was examined as well. According to the existing research, we made the following hypotheses:

Hypothesis 2: The post-pandemic entrepreneurial environment has a substantial effect on entrepreneurial self-efficacy.
Hypothesis 3: Students’ entrepreneurial self-efficacy has a significant influence on entrepreneurial intention.
Hypothesis 4: Entrepreneurial self-efficacy can serve as a mediator for the entrepreneurial intention in the post-pandemic entrepreneurial environment.

## Methods

### Participants and Procedures

The test was completed between August and October 2020 and was divided into a pre-test and a formal test. Complete data were tested and retrieved twice. Two questionnaires were administered to different participants.

#### Pre-test Sample Adopted in the Study

The pre-test questionnaire was accomplished in August 2020. 127 questionnaires were distributed to students who were majoring in foreign languages at a comprehensive university in Zhejiang Province, China with the convenience sampling method. The total number of participants is 127. Eight invalid questionnaires were excluded, and the efficiency of the questionnaire was thus 93.7%. Among them, 73 students majored in English, nine majored in Japanese, and 37 majored in German; 62 were juniors, 48 were seniors, and nine were postgraduates. After an analysis of the content, reliability, and validity of questionnaires, some items and revised the initial questionnaire was deleted to finalize the formal one.

#### Formal Sample Adopted in the Study

The official questionnaire was carried out from the end of August to October 2020. The questionnaires were distributed to students studying in three schools in Zhejiang Province, China with the stratified random sampling method. The three schools included a comprehensive university, a general private university, and a general junior college. With an online platform^1^, a total of 1,050 questionnaires were distributed and retrieved. To ensure that participants were represented, 350 individuals were drawn from each of the three schools based on four demographic variables: gender, profession, educational background, and family background, for achieving the integrity of the sample structure. Finally, 913 valid questionnaires (effective rate 86.95%) were obtained, of which 220 were completed by men and 693 were accomplished by women. Participants were students who are majoring in science, engineering, agriculture, and medicine (*n* = 134), humanities and social sciences (*n* = 186), sports and art (*n* = 453), and business administration (*n* = 140). Concerning participants’ educational background, 563 participants had a junior college degree, 313 had a bachelor’s degree, and 37 had a postgraduate degree. A total of 240 participants had a family member who had started their businesses, and 673 people did not. 374 participants were the only child in their family and 539 had siblings.

#### Test Process

This study was carried out in accordance with there commendations of the Human Ethics Committee of the Ningbo University. Class was designed to be the unit of the test, and one postgraduate student who had experience in this kind of testing before was responsible for the test. Before the test started, the researcher had provided specific training for the responsible person, which includes the instructions to the questionnaire, the content of the questionnaire, and notes of the testing process. During the test, the respondent would be informed of the academic purpose of the test and the anonymity of the questionnaire. The respondents were able to finish all the questions in the questionnaire with adjusted time, which is in line with their situation and can be more than enough.

### Measures

The initial questionnaire applied in this study included four sections and a total of 28 items, which includes seven items measuring the basic information of the participant, five items on the entrepreneurial self-efficacy scale, five items assessing entrepreneurial intention, and 11 items evaluating the post-pandemic entrepreneurial environment (including the four original items about the entrepreneurial environment and seven additional items related to the post-pandemic entrepreneurial environment). The questionnaire was also translated and revised for non-Chinese students.

The translation and revision of three scales were conducted in line with the following procedure: First, two master students majoring in English translation translated and back-translated questions of the original one to narrow the cross-cultural gap. Second, seven questions concerning the post-pandemic entrepreneurial environment were added to the entrepreneurial environment questionnaire and were reviewed by experts in this field to verify the questionnaire. Third, 10 college students were randomly selected to complete the questionnaire to make sure that there were no semantic ambiguities or unclear expressions. Fourth, 119 college students were selected for the pre-test with the convenience sampling method. Finally, the formal questionnaire was determined with item analysis and exploratory factor analysis achieved in the three scales of the pre-test questionnaire.

#### Background Variables

This part of the questionnaire collects data on students’ sex, major, grade, educational background, family members’ entrepreneurship history, whether they are from a one-child family or not, and a possible choice of entrepreneurship.

#### The Post-pandemic Entrepreneurial Environment Scale

This 11-item scale is a revised version based on the Entrepreneurial Environment Questionnaire (Jena, 2020). The revised questionnaire has seven questions. It is scored on a 5-point scale. Analysis of pre-test results shows: KMO = 0.832, Cronbach’s alpha = 0.877, and factor loading values of 0.721−0.911. This scale, therefore, owns good reliability.

#### The Entrepreneurial Intention of College Students Scale

The Questionnaire of the Entrepreneurial Intention of College Students is a 6-item unidimensional scale, which was designed by Li and Chen (2009). After revised by Jena (2020), there are five questions. And each of them is scored with a 5-point scale. The overall reliability of the pre-test scale is 0.918, with a KMO of 0.889 and factor loading values of 0.721 to 0.911. This scale, therefore, shows good reliability.

#### The Entrepreneurial Self-Efficacy Scale

The Entrepreneurial Self-Efficacy Questionnaire is designed by Wilson et al. (2007) and has been tested to have a good validity (Rajib and Niladri, 2020). It has five items, and each of them is scored with a 5-point scale. This scale also presents a good validity.

### Data Analysis

Reliability and validity of the scale of post-pandemic entrepreneurial environment, entrepreneurial self-efficacy, and entrepreneurial intention are assessed with SPSS and AMOS. And then, a correlation analysis is adopted to explore the relationship between the three main variables in SPSS. Finally, the specific mechanisms that can underlie these associations, including the mediating effect of entrepreneurial self-efficacy was examined, and the significance level was set at 0.05.

Cronbach’s alpha coefficient is applied as a reliability measure; Cronbach’s alpha is 0.959, 0.944, and 0.962 for the post-pandemic entrepreneurial environment scale, entrepreneurial self-efficacy scale, and entrepreneurial intention scale, respectively.

The validity of the questionnaire is tested with exploratory factor analysis and confirmatory factor analysis. The KMO values for the self-efficacy scale, the entrepreneurial intention scale, and the post-pandemic entrepreneurial environment scale are 0.95, 0.89, and 0.911, respectively, and all had a *p*-value < 0.001 (Table 1). Consequently, all three scales are considered to have good structural validity.

**TABLE 1:** Reliability and Validity of the Post-pandemic Entrepreneurial Environment, Entrepreneurial Self-Efficacy, and Entrepreneurial Intention Scales.

	**Values**	**PEE**	**ESE**	**EI**
Reliability	Cronbach’s alpha	0.959	0.944	0.962
	KMO Values	0.911	0.908	0.913
	Bartlett’s test of sphericity *P*-value	0.000	0.000	0.000
Validity	Interpretation Rate	80.45%	81.99%	86.93%
	Factor Loading Value	0.801∼0.927	0.88∼0.915	0.864∼0.932

***P < 0.001; PEE refers to Post-pandemic Entrepreneurial Environment; ESE means Entrepreneurial Self-Efficacy; EI stands for Entrepreneurial Intention.

AMOS is applied to carry out the confirmatory factor analysis, and the model adaptation is presented in Table 2. Based on the verification of various indicators, the three scales adopted in this study are viewed as one with good reliability and validity, which supports the report that they include accurate and effective questions and can collect reliable research data (Hou et al., 2004).

**TABLE 2:** Models of the Confirmatory Factor Analysis.

**Scales**	**RMSEA**	**RMR**	**GFI**	**CFI**	**IFI**	**CN (HOELTER. 05)**
	**(<0.08)**	**(<0.08)**	**(>0.9)**	**(>0.9)**	**(>0.9)**	**(>200)**
PEE	0.033	0.004	0.997	0.999	0.999	1071
ESE	0.074	0.01	0.98	0.993	0.993	272
EI	0.069	0.006	0.991	0.997	0.997	404

*PEE refers to Post-pandemic Entrepreneurial Environment; ESE means Entrepreneurial Self-Efficacy;EI stands for Entrepreneurial Intention.*

## Results

### Descriptive Statistics and Correlation Analysis

#### The Status Quo of Entrepreneurial Intention, Entrepreneurial Environment, and Self-Efficacy

As shown in Table 3, the mean total score is 3.86 (SD = 0.72) for the entrepreneurial environment scale, 3.49 (SD = 0.81) for the self-efficacy scale, and 3.45 (SD = 0.90) for students’ entrepreneurial intention scale. Given that all scales are measured with a 5-point scoring system, these results indicate that participants have scored at the upper-middle level. Besides, when asked “which type of entrepreneurship do you prefer in the post-pandemic era,” 60% of the students chose online business.

**TABLE 3:** The Post-pandemic Entrepreneurial Environment, Entrepreneurial Self-Efficacy, and Entrepreneurial Intentions.

**Variables**	**M**	**SD**	**1**	**2**
ESE	3.486	0.812		
PEE	3.456	0.909	0.670***	
EI	3.869	0.729	0.788***	0.682***

***P < 0.001; PEE refers to Post-pandemic Entrepreneurial Environment; ESE means Entrepreneurial Self-Efficacy; EI stands for Entrepreneurial Intention.

#### Differences in Entrepreneurial Intentions by Sex, Family Members’ Entrepreneurship History, and Being an Only Child or Not

Independent samples *t*-tests are rolled out with entrepreneurial intention as the dependent variable and sex (male/female), family members’ entrepreneurship history (yes/no), and one-child family (yes/no) as the independent variables. Male participants have significantly greater entrepreneurial intentions than female participants (*t* = 2.637, *p* < 0.01), and students whose family members had an entrepreneurship history are more likely to have greater entrepreneurial intention than students whose family members did not have an entrepreneurship history (*t* = 3.812, *p* < 0.01). There is no significant difference in entrepreneurial intention for whether students were the only child in the family or not.

An analysis of variance (ANOVA) is conducted as well with entrepreneurial intention as the dependent variable and the major and educational background as the independent variables. There are significant differences in entrepreneurial intention between students from different majors [*F*(4,913) = 19.555, *p* < 0.001]. Specifically, the entrepreneurial intention of students majoring in science, engineering, agriculture, and medicine is significantly higher than that of students majoring in humanities and social sciences (*p* < 0.001), and the entrepreneurial intention of students majoring in sports and art is significantly higher than that of students majoring in humanities and social sciences (*p* < 0.001) as well as in business administration (*p* < 0.01). Furthermore, educational background has a vital influence on entrepreneurial intentions [*F*(3,913) = 64.314, *p* < 0.001], whereby junior college students have higher entrepreneurial intentions than undergraduates. However, the number of graduate students is too small to be considered.

#### The Post-pandemic Entrepreneurial Environment, Entrepreneurial Self-Efficacy, and Entrepreneurial Intentions

Table 3 represents the averages, standard deviations, and correlation coefficients for the post-pandemic entrepreneurial environment, entrepreneurial self-efficacy, and entrepreneurial intention. College students’ perception of the post-pandemic entrepreneurial environment and entrepreneurial self-efficacy is not obvious (M1 = 3.486, M2 = 3.456), which is slightly higher than the expected average of 3, but the entrepreneurial intention is relatively high (M3 = 3.869), close to 4. Entrepreneurial self-efficacy is positively correlated with both the post-pandemic entrepreneurial environment and entrepreneurial intention (Table 3).

### Structural Equation Modeling Test

After analysis on the overall reliability and validity of the three scales, AMOS22.0 is adopted to construct a structural equation modeling, which generates a model with path coefficients, performing analysis on path effects. This step is applied to verify Hypothesis 1, Hypothesis 2, and Hypothesis 3.

#### Model Generation

**FIGURE 1:**
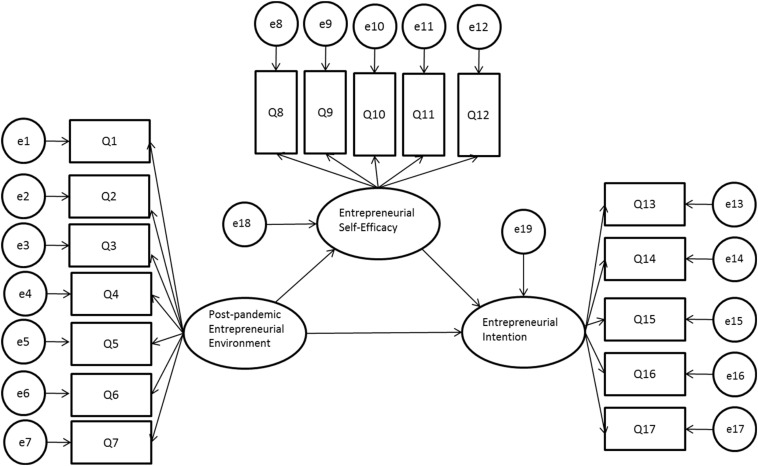
The Structural Equation Modeling Diagram of the post-pandemic entrepreneurial environment scale, entrepreneurial self-efficacy scale, and entrepreneurial intention scale (Q1∼Q17 represent the item of the scale, e1∼e17 refer to the error terms of the items).

To present the relationship between the post-pandemic entrepreneurial environment, entrepreneurial self-efficacy, and entrepreneurial intention scale scores, a structural equation modeling diagram (Figure 1) is formed based on the assumptions of this study.

#### Structural Equation Modeling Fitting Analysis

With analysis on the structural equation modeling diagram of the post-pandemic entrepreneurial environment scale, entrepreneurial self-efficacy scale, and entrepreneurial intention scale, the results of the model fit are presented in Table 4. Most of the indicators can meet the ideal standard (RMSEA = 0.077, RMR = 0.038, GFI = 0.909, CFI = 0.968, IFI = 0.968, PNFI = 0.806), and the model fits well on the whole, which indicates that the hypotheses can be established.

**TABLE 4:** The Overall fitting situation of the Model.

**Model Fit**	**RMSEA**	**RMR**	**GFI**	**CFI**	**IFI**	**PNFI**
Standard	<0.08	<0.08	>0.9	>0.9	>0.9	>0.5
Results	0.077	0.038	0.909	0.968	0.968	0.806

#### Analysis of Model Path

AMOS is applied to run the structural equation model based on the collected data (Table 5). The effect of the post-pandemic entrepreneurial environment on entrepreneurial intention is significant and positive, with a coefficient of 0.236. The influence of the post-pandemic entrepreneurial environment on entrepreneurial self-efficacy is also vital and positive, with a coefficient of 0.696. The impact of entrepreneurial self-efficacy on entrepreneurial intention is significantly positive as well, with a coefficient of 0.654. Besides, the post-pandemic entrepreneurial environment had a significant positive effect on entrepreneurial self-efficacy, with a coefficient of 0.654.

**TABLE 5:** Coefficients of the Model Path Analysis.

**Relationships**	**Standardized path coefficient**	**Standard error**	**Critical ratio**
ES ← PEE	0.696***	0.031	22.293
EI← PEE	0.236***	0.036	7.73
EI← ESE	0.654***	0.039	19.311

PEE refers to Post-pandemic Entrepreneurial Environment; ESE stands for Entrepreneurial Self-Efficacy; EI represents Entrepreneurial Intention; ***P < 0.001.

#### Bootstrap’s Mediating Effect Analysis

With the analysis on the relationship between post-pandemic entrepreneurial environment, entrepreneurial self-efficacy, and entrepreneurial intentions, the specific mechanisms on how these variables interact, as well as the mediating effects of entrepreneurial self-efficacy is explored, which is applied to verify Hypothesis 4.

In the analysis of the indirect effects, MacKinnon et al. (2004) have compared the traditional method, distribution of the product, and five nonparametric bootstrap methods, and found that the bias-corrected nonparametric percentile bootstrap method can provide the most accurate confidence intervals and the highest statistical power for measuring the mediating effects. A bootstrap method is adopted in AMOS to test indirect effects.

The effect values of the post-pandemic entrepreneurial environment on entrepreneurial intentions, which are obtained with the nonparametric percentile bootstrap method, are shown in Table 6. The confidence intervals of the bias-corrected non-parametric percentiles for the total effect, direct effect, and indirect effect do not include zero, which signifies that the post-pandemic entrepreneurial environment has a significant total and direct impact on entrepreneurial intentions. Furthermore, the indirect effect of entrepreneurial self-efficacy is also substantial, with an effect size between 0.642 and 0.735; the direct effect size is between 0.166 and 0.319, and the indirect effect size ranges between 0.392 and 0.524. Entrepreneurial self-efficacy brings a partially indirect effect on the post-pandemic entrepreneurial environment and entrepreneurial intentions, accounting for 65.9% of the total effect.

**TABLE 6:** The Effect of Post-pandemic Entrepreneurial Environment on Entrepreneurial Intentions.

**EI ← PEE**	**Path coefficients**	**95% CI**
Total Effect	0.690***	0.735, 0.642
Direct Effect	0.236***	0.319, 0.166
Indirect Effect	0.455***	0.524, 0.392

PEE refers to Post-pandemic Entrepreneurial Environment; EI stands for Entrepreneurial Intention; ***P < 0.001.

## Discussion and Conclusion

### In the Post-pandemic Era, College Students Still Have Entrepreneurial Intentions and Are More Inclined to Start an Online Business

The results of this study show that college students still have entrepreneurial intentions in the post-pandemic world. In a sample survey of 2,975 college students, Yan and Ye (2009) have found a medium level of entrepreneurial intent. As a result, compared with pre-epidemic studies, the post-pandemic environment has posed little effect on the entrepreneurial intentions of college students, which suggests that while the epidemic may harm the country’s economy, it also witnesses many favorable entrepreneurial policies that are put in place to support enterprises during these difficulties. Besides, the questionnaire reveals that college students in the post-pandemic environment are inclined to start a business online compared with that in the pre-epidemic environment. This may be caused by the changes in the social environment after the pandemic. For instance, people may be more likely to buy products online and engage in online education, and their way of thinking can be more adaptable to the Internet environment. The post-pandemic entrepreneurial environment is both an opportunity and a challenge for college students. As an emerging group, college students are more familiar with the Internet than preceding generations with a high degree of acceptance, which shows that starting a business online can become the main way to guide entrepreneurial intention, as reflected in the results.

### Sex, Family Members’ Entrepreneurship History, Major, and Educational Background’s Impact on Students’ Entrepreneurial Intentions

It is found in this study that the entrepreneurial intentions of male students are significantly higher than that of female students, which is consistent with the conclusion of previous studies (Zhu and Zhou, 2010; Li et al., 2011). This can be related to the conventional idea in Chinese culture that “men should be outgoing and start a career and women should work hard to deal with home errands.” In the embodiment of these gender traits, men in China are more likely to be trained as one with toughness and bravery, while women in China are more likely to be taught as who are quiet and careful.

Second, our results show that students whose family members have an entrepreneurship history have significantly greater entrepreneurial intentions than those who do not, which may be caused by the family entrepreneurial atmosphere. Students whose family members have an entrepreneurship history have more opportunities to observe and learn actual entrepreneurial practices; indeed, a person can learn new responses by observing the behavior of others and reinforcing the results according to Bandura’s social cognitive theory. Therefore, students whose family members have entrepreneurial experiences are more likely to be familiar with entrepreneurship and show more entrepreneurial intentions than students whose family members do not.

In addition, significant differences in entrepreneurial intentions are also found between various majors, in which students majoring in science, technology, agriculture, and medicine have significantly higher entrepreneurial intentions than those majoring in humanities and social sciences, and students majoring in sports and art have significantly higher entrepreneurial intentions than those majoring in humanities and social sciences. This finding can be explained by the demand of the domestic market. Most students majoring in science, engineering, agriculture, medicine, sports, and art have links with more professional technology and larger market demand, which makes them easier to start a business.

Finally, students with a junior college degree have stronger entrepreneurial intention than those with a bachelor’s degree, and the reason behind this may be that junior colleges place greater focus on practice and application compare with bachelor’s degree programs.

### The Links Between Post-pandemic Entrepreneurial Environment, Entrepreneurial Self-Efficacy, and Entrepreneurial Intention

The results illustrate that the influence of the post-pandemic entrepreneurial environment on entrepreneurial intention and entrepreneurial self-efficacy is rather vital and positive, and entrepreneurial self-efficacy can also greatly affects entrepreneurial intention. The results show that the better the post-pandemic entrepreneurial environment, the stronger the entrepreneurial intention, which is consistent with previous reports (Zhu and Zhang, 2014; Ye and Fang, 2017). It is found in this study that the better the entrepreneurial environment, the stronger the self-efficacy and entrepreneurial intention, which is also consistent with the social cognitive theory, which states the influence of environmental and personal factors on behavior. When the post-pandemic entrepreneurial environment is sound and stable, more successful entrepreneurs will emerge, people’s entrepreneurial self-efficacy will increase, and more people will be motivated to develop entrepreneurial intentions.

### Entrepreneurial Self-Efficacy Have a Strong Mediating Effect on the Impact Caused by the Post-pandemic Entrepreneurial Environment on Entrepreneurial Intentions, and the Mediating Effect Is Relatively High

In this study, it is safe to conclude that the stronger an individual’s self-efficacy, the stronger their entrepreneurial intention; the better the environment for entrepreneurship, the stronger the entrepreneurial intentions, which further validates the ternary interaction theory on the links between an individual, their environment, and behavior, as proposed by the social cognitive theory. At the same time, it is also found that self-efficacy has a strong mediating effect on the impact brought by the post-pandemic entrepreneurial environment on entrepreneurial intentions, which further illustrates that despite the global economic trauma brought by the epidemic, the human factor should never be ignored. In the post-pandemic entrepreneurial environment, the role of entrepreneurial self-efficacy is still critical.

## Suggestions

### Further Optimizing the Entrepreneurial Environment for College Students in the Post-pandemic Era

First, the situation of the epidemic should never be overlooked and mild measures need to be continued to provide a safe environment for the public. Especially, the current situation is still unstable with possible further outbreaks. Second, it is vital to maintain stable domestic economic growth. Finally, the government should consider rolling out more entrepreneurial policies to support the entrepreneurship of college students.

### Entrepreneurship Education in Universities and Colleges Should Strengthen Students’ Entrepreneurial Self-Efficacy

Entrepreneurship education can enhance students’ self-efficacy through both curricular and extra-curricular activities. For example, students’ skills for problem-solving and money management can be cultivated and developed at both classroom and amateur practice; skills in communication and persuasion can be also fostered through speech competitions, roadshows, and other activities; and leadership and independent spirit can be encouraged through participation in student organizations, such as student councils, clubs, as well as entrepreneurship associations.

### Schools Entrepreneurship Education Should Stratify and Classify College Students According to Their Abilities

Students with family members who have started their businesses or majors with a large market demand should be paid extra attention to develop their entrepreneurial skills. The process of entrepreneurship education needs to be considered in conjunction with majors to encourage students to practice their professional knowledge so as to start their businesses. Besides, universities can set up an on-campus entrepreneurship park to provide opportunities for students who are interested in starting their businesses, and the school can also look for successful alumni entrepreneurs to do lectures so that students can know the exemplary entrepreneurs around them.

## Limitations and Future Research Directions

In this study, questionnaires are adopted to collect data and conduct analysis with a focus on the influence of the post-pandemic entrepreneurial environment and entrepreneurial self-efficacy on entrepreneurial intentions. However, the study leaves the question of whether other factors might also influence entrepreneurial intentions unanswered. Future research can supplement the data collected in this study to better understand the challenges affecting entrepreneurial intentions in the post-pandemic environment, and different independent variables can be included accordingly. Such investigations can help to enhance the entrepreneurial intentions of college students in the post-pandemic era.

## Data Availability Statement

The original contributions presented in the study are included in the article/supplementary material, further inquiries can be directed to the corresponding author/s.

## Ethics Statement

This study was carried out in accordance with the recommendations of the Human Ethics Committee of the Ningbo University. The patients/participants provided their written informed consent to participate in this study.

## Author Contributions

JZ designed the study, analyzed the data, and drafted the manuscript. JH assisted in analyzing and interpreting the data, and participated in the revision of the manuscript. Both authors contributed to the study and approved the submitted version.

## Conflict of Interest

The authors declare that the research was conducted in the absence of any commercial or financial relationships that could be construed as a potential conflict of interest.

## Footnotes

1 www.wjx.cn
